# High molecular risk variants, severe thrombocytopenia and large unstained cells count affect the outcome in primary myelofibrosis

**DOI:** 10.1007/s13353-023-00771-x

**Published:** 2023-07-29

**Authors:** Zuzanna Kanduła, Michał Janowski, Barbara Więckowska, Edyta Paczkowska, Aleksandra Mroczkowska-Bękarciak, Marta Sobas, Krzysztof Lewandowski

**Affiliations:** 1 Department of Hematology and Bone Marrow Transplantation, Poznań University of Medical Sciences, Poznan, Poland; 2 Department of General Pathology, Pomeranian Medical University, Szczecin, Poland; 3 Department of Computer Science and Statistics, Poznań University of Medical Sciences, Poznan, Poland; 4 Department of Hematology, Blood Neoplasms and Bone Marrow Transplantation, Medical University, Wrocław, Poland

**Keywords:** Primary myelofibrosis, High molecular risk variants, Large unstained cells count, Platelet count, *ASXL1*, *U2AF1*

## Abstract

Apart from the driver mutations, high molecular risk (HMR) variants and other factors have been reported to influence the prognosis of primary myelofibrosis (PMF). The aim of our study was to investigate the impact of laboratory and molecular characteristics at the time of diagnosis (TOD) on the PMF outcome. The study group consisted of 82 patients recruited from three Polish university centers. Among the driver mutations, only *CALR* type 1 positively influenced the overall survival (OS). The risk of progression to accelerated or blastic disease phase (AP/BP) did not depend on the driver mutation type, but was closely associated with the presence of HMR variants (*p* = 0.0062). The risk of death (ROD) was higher in patients with HMR variants (OR[95%CI] = 4.33[1.52;12.34], *p* = 0.0044) and in patients with a platelet count at the TOD between 50–100 G/L (HR[95%CI] = 2.66[1.11;6.35]) and < 50 G/L (HR[95%CI] = 8.44[2.50;28.44]). Median survival time was 7.8, 2.2 and 1.4 years in patients with large unstained cells (LUC) count of [0.0–0.2], (0.2–0.4] and > 0.4 G/L at the TOD, respectively. We found an unexpected, hitherto undescribed, association between LUC count at the TOD and PMF prognosis. Our analysis led to the following conclusions: in PMF patients at the TOD 1) the presence of HMR variants, especially combined, is associated with an increased risk of progression to the AP and BP, and shorter OS, 2) severe thrombocytopenia confers worse prognosis than the moderate one, 3) LUC count is closely related with the disease phase, and associated with the ROD and OS.

**Supplementary Information:**

The online version contains supplementary material available at 10.1007/s13353-023-00771-x.

## Introduction

Primary myelofibrosis (PMF) together with polycythemia vera (PV) and essential thrombocythemia (ET) are classified as a classical Philadelphia negative myeloproliferative neoplasm (MPN Ph-) (Khoury et al. 2022). The annual incidence of PMF is estimated at 0.47 (0.22–0.99) per 100.000 and is slightly higher for males than for females (0.59 vs. 0.30) (Titmarsh et al. 2014). PMF occurs at all ages, but is most common around the seventh decade of life (Moulard et al. 2014; Penna et al. 2019; Shallis et al. 2020). Fatigue, fever, weight loss and night sweats are typical PMF symptoms, associated with progressive anemia, thrombocytopenia and hepato/splenomegaly. The disease outcome is complicated by unprovoked thrombosis, frequently in patients receiving anti-thrombotic or cytoreductive treatment (Tefferi 2005; Tefferi et al. 2011; Hernández-Boluda et al. 2022).

A large number of laboratory abnormalities, including variable degrees of megakaryocyte atypia, reticulin and/or collagen bone marrow fibrosis, ineffective erythropoiesis, increased angiogenesis, extramedullary hematopoiesis, and abnormal cytokine expression resembling chronic inflammation, are also characteristic for PMF. Over the disease course, normal bone marrow tissue is gradually replaced with fibrous scar-like material, leading to progressive bone marrow failure (Kuter et al. 2007). The diagnostic criteria of PMF have been changed during the last 15 years, with new ones formulated by the WHO in 2016 and maintained in 2022 (Arber et al. 2016; Khoury et al. 2022).

Molecular landscape of driver mutations is well established and includes *JAK2*V617F, *CALR* and *MPL,* with the frequency of 50–60%, 25–30%, 5–10%, respectively. About 10% of patients do not have any identifiable driver mutations and are classified as triple negative (TN). Among MPN, PMF has the worst prognosis, with an estimated median survival of 3.6–6.5 years and an estimated 5-year relative survival of about 40% (Cervantes et al. 2012; Tefferi et al. 2014a; Shallis et al. 2020). However, the data indicate that the overall survival (OS) and risk of leukemic transformation strongly depend on the driver mutation type (Tefferi et al. 2014a, b; Rumi and Cazzola 2017). Rumi et al. reported that median OS was 17.7 years in *CALR*-mutated, 9.2 years in *JAK2*-mutated, 9.1 years in *MPL*-mutated, and 3.2 years in TN patients (Rumi and Cazzola 2017). The cumulative incidence of PMF progression to the blastic phase (BP) has been reported as 0.129–0.142 for PMF, significantly higher than in the case of ET and PV (0.038 and 0.068, respectively) (Tefferi et al. 2014a; Vallapureddy et al. 2019). The leukemic transformation risk is higher in the TN (HR[95%CI] = 7.6[2.8;20.2]) and *JAK2* positive (HR[95%CI] = 2.7[1.1;6.6]) patients than in the *CALR*-mutated ones. A similar analysis performed in the case of *MP*L-mutated individuals (HR[95%CI] = 1.9[0.5;7.7]) showed no differences (Tefferi et al. 2014a).

Apart from driver mutations, non-driver variants have an impact on the PMF long-term prognosis and outcome, as well. Several prognostic models have been proposed for PMF patients, including International Prognostic Scoring System (IPSS) (Cervantes et al. 2009), Dynamic International Prognostic Scoring System (DIPPS) (Passamonti et al. 2010), Mutation-Enhanced International Prognostic Scoring System 70/70 + (MIPSS70/70 +) (Tefferi et al. 2018b) and Genetically Inspired Prognostic Scoring System (GIPSS) (Tefferi et al. 2018c). GIPSS stratifies PMF patients by mutations and karyotype solely, whereas MIPSS bases on the clinical features and mutations landscape, especially in genes *ASXL1, SRSF2, EHZ2, IDH1, IDH2, U2AF1*, defined as high molecular risk (HMR) mutations/variants (Tefferi et al. 2018c, b).

The aim of our study was to investigate the impact of chosen HMR genetic variants (*ASXL1* exon 13, *SRSF2* exon 1, *U2AF1* exon 2 and 6, *IDH1* exon 4, *IDH2* exon 4) on the clinical manifestation and outcome of PMF patients diagnosed and treated in our centers during the last 10 years. Special attention was paid to the relation between the laboratory disease characteristics at the time of diagnosis (TOD) and long-term PMF outcome, especially in terms of the frequency of progression to more advanced phases and death, dependently on the laboratory characteristic of individual cases. Although *U2AF1*S34 is not classified as a HMR variant (Tefferi et al. 2018a), we analyzed *U2AF*1S34 together with other HMR variants, as it was reported as pathogenic in PMF (Tamari et al. 2019) and in myelodysplastic syndromes, as well (Li et al. 2020).

### Study group characteristics

The study group consisted of 82 pts recruited to the study between 2012 and 2021 from the three Polish University centers – the Department of Hematology and Bone Marrow Transplantation of Poznań University of Medical Sciences in Poznań, the Department of Hematology of Pomeranian Medical University in Szczecin and Department of Hematology, Blood Neoplasms and Bone Marrow Transplantation of Medical University in Wrocław. The diagnosis of PMF was established according to the WHO criteria applicable at the TOD – 2008 or 2016, respectively (Vardiman et al. 2009; Arber et al. 2016), and verified according to the WHO 2016 criteria at the study entry. The grade of the bone marrow fibrosis was assessed according to the European Consensus on grading bone marrow fibrosis and the assessment of cellularity (Thiele et al. 2005). Patient results and medical history were analysed to exclude misdiagnosis of post-PV-MF, post-ET-MF and ET in the case of pre-fibrotic PMF. Bone marrow samples were assessed at the TOD and, thereafter, if clinical or laboratory symptoms of the disease progression to a more advanced disease phase were noted. The general characteristics of the studied patients are presented in Table 1.

**Table 1:** General characteristics of the PMF patients studied

Studied group	*n* = 82 (%)
Male/female	53/29 (65/35)
Median age at time of diagnosis, y [range]	63 [25–89] (-)
IPSS	
Low	15 (18)
Intermediate-1	12 (15)
Intermediate-2	30 (37)
High	25 (30)
*JAK2*V617F positive	49 (60)
*CALR* positive:	19 (23)
*CALR* type 1	15 (18)
*CALR* type 2	3 (4)
*CALR* other type	1 (1)
*MPL* positive	5 (6)
Triple negative	9 (11)
Patients carrying high molecular risk variants:	31 (38)
1 variant	24 (29)
2 variants	7 (9)

## Materials and methods

DNA was extracted from whole-blood leukocytes at the TOD or first evaluation at our Department using QIAmp DNA Mini Kit (Qiagen). The assessment for the presence of the *JAK2*V617F mutation was conducted by quantitative allele-specific RQ-PCR according to Larsen et al. (Larsen et al. 2007), standardized by cooperation with MPN&MPNr EuroNet (Jovanovic et al. 2013). High resolution melt analysis (HRMA) was used to detect the following variants: *CALR* exon 9, *MPL* exon 10, *SRSF2* exon 1, *U2AF1* exon 2 and 6, *IDH1* exon 4, *IDH2* exon 4. For the identification of the variant type screened by HRMA, Sanger sequencing was applied, using the BigDye Terminator v3.1 Cycle Sequencing kit (Applied Biosystems, Thermo Fisher Scientific). Similarly, Sanger sequencing was applied for exon 13 (range Ile574 to Glu727) in the *ASXL*1 gene analysis (a region covering at least 83% of all known *ASXL1* mutations) (Gelsi-Boyer et al. 2009; Pratcorona et al. 2012). The PCR primer sequences used and the details of the method applied are listed in Table S1. Complete blood count (CBC), including large unstained cells (LUC) count, was performed using high-volume hematology analyzer Advia 2120i® (Siemens).

## Statistical analysis

Nominal data were described using counts and percentages for each category. A comparison of such data between the study and control group was performed using the chi-square test or its correction (Fisher's exact test), whenever the numbers in individual categories were too low. To describe the magnitude of the obtained effect, an odds ratio (OR) was determined together with a 95% confidence interval (CI), giving the chance of occurrence of the event in the exposed group in relation to the reference category. Continuous data were described using mean ± standard deviation and median with quartiles.

The receiver operating characteristic (ROC) curve was used to find the best cut-off point for the continuous variables that would allow high sensitivity and specificity for predicting death. The size of the area under the curve was determined and its significance assessed using DeLong's method. In places where an area significantly greater than 0.5 was obtained, a cut-off point was determined using the Youden Index. The sensitivity and specificity obtained for this point are also given. The assessment of the association of individual variables with survival time was performed using Cox proportional hazards regression models; selected categorical data were also assessed using the log-rank test and presented graphically using the Kaplan–Meier curve. In order to test whether the relationships found were independent of the collected co-variates, the Cox proportional hazards regression model was extended into two steps. In the first one, individual variables were adjusted for gender and age – the minimally adjusted model, and in the second one, for those co-variates that were statistically significant in the univariate analysis, had no missing data and were not redundant (Pearson correlation coefficient less than 0.5) – the fully adjusted model. The resulting sizes were described by the Hazard Ratio together with the 95% confidence intervals. All analyses were performed in PQStat v1.8.4 software. The significance level of 0.05 was assumed.

## Results

### Driver mutation status of the studied patients

**Fig. 1:**
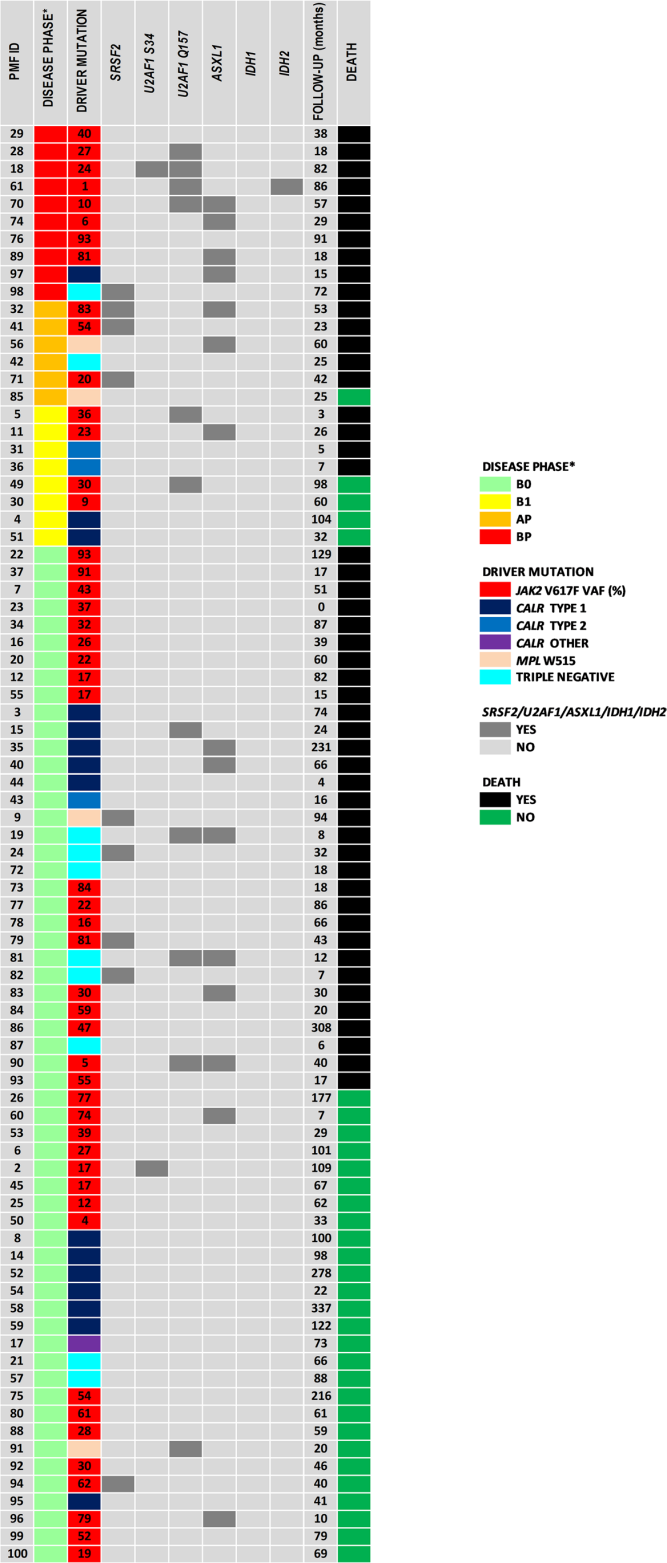
Detailed molecular characteristics of the PMF studied patients. * The highest blasts percentage in the peripheral blood/bone marrow (PB/BM) noticed during the disease outcome was used for the analysis: B0 [0–5%), B1 [5–10%), AP [10–19%] and BP ≥ 20%. Abbreviations: AP – accelerated phase, BP – blast phase

Among the 82 studied individuals with a proven diagnosis of PMF according to the WHO 2016 criteria, 49 (60%) were *JAK2*V617F, 15 (18%) *CALR* type 1, 3 (4%) *CALR* type 2 and 5 (6%) *MPL* positive. Another *CALR* type mutation was confirmed in one (1%) patient. Nine other patients (11%) were TN (Table 1). Detailed molecular characteristics of the studied patients are presented in Fig. 1. All the patients studied were diagnosed with the chronic PMF phase.

### High molecular risk variants status of the studied patients

The overall frequency of the HMR variants (*ASXL1*, *SRSF2*, *U2AF1*, *IDH2*) was 38% (31/82) and 39%, 21%, 80% and 44%, according to *JAK2*V617F, *CALR*, *MPL* mutation and TN status, respectively. In 24/82 (29%) patients, a single HMR variant was detected. In 7 other patients, the coexistence of two HMR variants was confirmed. Among the studied coexisting genetic variants, the *ASXL1* mutations were the most frequent. None of the analyzed patients carried the *IDH1* mutation. The correlation between the coexistence of *JAK2*V617F and *SRSF2* mutations was found (C-Pearson adjusted = 0.42, *p* = 0.0353) (Table 2).

**Table 2:** The frequency of the HMR variants according to the driver mutation in PMF patients

			HMR variant type									
	HMR variant presence		*SRSF2*		*U2AF1*S34		*U2AF1*Q157		*ASXL1*		*IDH2*	
Driver mutation n (%)	No	Yes	No	Yes	No	Yes	No	Yes	No	Yes	No	Yes
*JAK2*V617F	30 (59)	19 (61)	44 (60)	5 (56)	47 (59)	2 (100)	42 (59)	7 (64)	40 (60)	9 (60)	48 (59)	1 (100)
*CALR*	15 (29)	4 (13)	19 (26)	0 (0)	19 (24)	0 (0)	18 (25)	1 (9)	16 (24)	3 (20)	19 (23)	0 (0)
*MPL*	1 (2)	4 (13)	3 (4)	2 (22)	5 (6)	0 (0)	4 (6)	1 (9)	4 (6)	1 (7)	5 (6)	0 (0)
*TN*	5 (10)	4 (13)	7 (10)	2 (22)	9 (11)	0 (0)	7 (10)	2 (18)	7 (10)	2 (13)	9 (11)	0 (0)
total	51	31	73	9	80	2	71	11	67	15	81	1
C-Pearson (adjusted)		0.38	0.42		0.18		0.21		0.07		0.13	
Fisher exact test (*p*-value)		0.1027	0.0353		1.0000		0.4085		1.0000		1.0000	

*Abbreviations*: *HMR* high molecular risk

The frequency of particular HMR variants in the *JAK2*, *CALR*, *MPL* positive and TN groups harboring HMR variants is shown in Table 3. In the *JAK2*V617F positive, *MPL* positive, and TN patients, the most common HMR variant was *SRSF2*, with the frequency of 49%, 80%, 67%, respectively. In the majority (75%) of *CALR*-mutated patients, the *ASXL1* variant presence was confirmed.

**Table 3:** The frequency of particular genetic variants in the *JAK2*, *CALR*, *MPL* positive, and TN PMF patients, harboring specific HMR variants

Driver mutation	Number of HMR variants	*SRSF2* n (%)	*U2AF1S34* n (%)	*U2AF1Q157* n (%)	*ASXL1* n (%)	*IDH2* n (%)
*JAK2*V617F	24	5 (21)	2 (8)	7 (29)	9 (38)	1 (4)
*CALR*	4	0 (0)	0 (0)	1 (25)	3 (75)	0 (0)
*MPL*	4	2 (50)	0 (0)	1 (25)	1 (25)	0 (0)
*TN*	6	2 (33)	0 (0)	2 (33)	2 (33)	0 (0)

*Abbreviations*: *HMR* high molecular risk

The *SRFS2* variant presence was confirmed in 9 out of 82 patients (11%). Eight out of 9 (89%) individuals carrying the *SRSF2* variant at the TOD died during the study outcome – 3 due to progression to the accelerated phase (AP) and one to the BP. 4 out 11 (36%) patients carrying the *U2AF1*Q157 mutation transformed to the BP. *U2AF1*S34 variant was detected in two cases: in the first patient as coexisting with the *U2AF1*Q157 variant (the patient progressed to the BP and died), and in the second patient who successfully underwent allogeneic stem cell transplantation and is still alive.

The *ASXL1* variant was found in 15/82 (18%) patients. Only two of them are still alive (the follow-up time is 7 and 10 months, respectively). The *IDH2* mutation was found in a *JAK2*V617F (VAF = 1%) positive patient carrying also the *U2AF1*Q157 mutation. Interestingly, after 3 years he progressed to the BP and died.

### Mutational status at the TOD and the disease phase

The disease evolution to the more advanced phases (AP or BP) was monitored during the patient follow-up. The highest blast percentage in the peripheral blood/bone marrow (PB/BM) noticed during the disease outcome was used for the final analysis. The following categories were formulated for the statistical assessment of the patients studied: B0 [0–5%), B1 [5–10%), AP [10–19%] and BP ≥ 20%. The frequency of the HMR variants coexistence and the number of variants per patient was 73%, 71%, 38%, 27% and 1.00, 0.86, 0.38, 0.32 in the BP, AP, B1 and B0, respectively (Table 4). The majority of patients (9/11, 82%) progressing to the BP was *JAK2*V617F positive.

**Table 4:** The frequency of HMR variants according to the disease phase

Disease phase^a^	Number of patients	HMR variants [n of patients] (%)	HMR variants [n of variants]	Non-variant per patient	*JAK2* n (%)	*CALR* n (%)	*MPL* n ( %)	*TN* n (%)
BP	11	8 (73)	11	1.00	9 (82)	1 (9)	0 (0)	1 (9)
AP	7	5 (71)	6	0.86	3 (43)	1 (14)	2 (29)	1 (14)
B1	8	3 (38)	3	0.38	4 (50)	4 (50)	0 (0)	0 (0)
B0	56	15 (27)	18	0.32	33 (59)	13 (23)	3 (5)	7 (13)

*Abbreviations*: *HMR* high molecular risk, *AP* accelerated phase, *BP* blastic phase

^a^ The highest blast percentage in the peripheral blood/bone marrow (PB/BM) noticed during the disease outcome was used for the analysis: B0 [0–5%), B1 [5–10%), AP [10–19%] and BP ≥ 20%

The risk of progression to a more advanced disease phase was closely associated with the presence of the HMR variants (*p* = 0.0062) and did not depend on the driver and HMR variant type, if the HMR variants were analyzed separately. The study of the impact of specific HMR variants on the disease progression to more advanced phases showed inconclusive results (Table 5).

**Table 5:** The risk of PMF progression to more advanced phases according to the presence of specific variants

			*p*-value	OR	−95%CI	+ 95%CI
Disease phase^a^						
	*JAK2*V617F					
	no (%)	yes (%)				
			0.3309 ^b^			
B0	23 (70)	33 (67)		reference		
B1	4 (12)	4 (8)		0.70	0.16	3.08
AP	4 (12)	3 (6)		0.52	0.11	2.56
BP	2 (6)	9 (18)		3.14	0.62	15.88
	*CALR*					
	no (%)	yes (%)				
			0.2246 ^b^			
B0	43 (68)	13 (68)		reference		
B1	4 (6)	4 (21)		3.31	0.72	15.10
AP	6 (10)	1 (5)		0.55	0.06	5.01
BP	10 (16)	1 (5)		0.33	0.04	2.83
	*MPL*					
	no (%)	yes (%)				
			0.1198 ^b^			
B0	53 (69)	3 (60)		reference		
B1	8 (10)	0 (0)		0.00	0.00	NA
AP	5 (7)	2 (40)		7.07	0.95	52.77
BP	11 (14)	0 (0)		0.00	0.00	NA
	TN					
	no (%)	yes (%)				
			0.8621 ^b^			
B0	49 (67)	7 (78)		reference		
B1	8 (11)	0 (0)		NA	NA	NA
AP	6 (8)	1 (11)		1.17	0.12	11.18
BP	10 (134)	1 (11)		0.70	0.08	6.34
	HMR variants					
	no (%)	yes (%)				
			0.0062 ^b^			
B0	41 (80)	15 (48)		reference		
B1	5 (10)	3 (10)		1.64	0.35	7.72
AP	2 (4)	5 (16)		6.83	1.20	39.06
BP	3 (6)	8 (26)		7.29	1.71	31.16
	*SRSF2*					
	no (%)	yes (%)				
			0.0702			
B0	51 (70)	5 (56)		reference		
B1	8 (11)	0 (0)		NA	NA	NA
AP	4 (6)	3 (33)		7.65	1.32	44.30
BP	10 (14)	1 (11)		1.02	0.11	9.69
	*U2AF1 S34*					
	no (%)	yes (%)				
			0.5363 ^b^			
B0	55 (69)	1 (50)		reference		
B1	8 (10)	0 (0)		NA	NA	NA
AP	7 (9)	0 (0)		NA	NA	NA
BP	10 (13)	1 (50)		5.50	0.32	95.32
	*U2AF1 Q157*					
	no (%)	yes (%)				
			0.0439 ^b^			
B0	51 (72)	5 (45)		reference		
B1	6 (9)	2 (18)		3.40	0.54	21.52
AP	7 (10)	0 (0)		NA	NA	NA
BP	7 (10)	4 (36)		5.83	1.26	27.02
	*ASXL1*					
	no (%)	yes (%)				
			0.0604 ^b^			
B0	49 (73)	7 (47)		reference		
B1	7 (11)	1 (7)		1.00	0.11	9.39
AP	4 (6)	3 (20)		5.25	0.97	28.55
BP	7 (10)	4 (27)		4.00	0.93	17.25
	*IDH2*					
	no (%)	yes (%)				
			0.3170 ^b^			
B0	56 (69)	0 (0)		reference		
B1	8 (10)	0 (0)		NA	NA	NA
AP	7 (9)	0 (0)		NA	NA	NA
BP	10 (12)	1 (100)		NA	NA	NA

*Abbreviations*: *OR* odds ratio, *CI* confidence intervals, *NA* not applicable, *AP* accelerated phase, *BP* blastic phase

^a^ The highest blast percentage in the peripheral blood/bone marrow (PB/BM) noticed during the disease outcome was used for the final analysis:B0 [0–5%), B1 [5–10%), AP [10–19%] and BP ≥ 20%

^b^ Fisher exact test

### HMR variants’ coexistence and the risk of death (ROD)

50 out of 82 (61%) analyzed PMF patients died during the study outcome (the median follow-up of 2.6 vs. 5.5 years in patients who died and are alive, respectively). In patients who died, the frequency of the HMR variants coexistence was higher than in the living patients (50 vs. 20%, the index of HMR variant per patient was 0.64 vs 0.19, respectively). The ROD was 4.33 times (OR[95%CI] = 4.33[1.52;12.34], *p* = 0.0044) and 5.27 times (OR[95%CI] = 5.27[1.10;25.98], p = 0.0381) higher in patients carrying the HMR variant and *ASXL1* variant at the TOD. There was no association between the ROD and the type of HMR variant other than *ASXL1*, if they were analyzed separately (Table S2). Also, no association between *JAK2*V617F VAF at the TOD, disease phase and the risk of pre-term death was found (Table S3).

### Analysis of other factors influencing the OS of PMF patients

Cox proportional hazard model (Table S4) showed that the age at the TOD was related to the OS time, regardless of sex and other variables that have also shown a significant relationship with the survival time. The ROD for the PMF patients studied increased by about 7% with each year; HR [95% CI] = 1.07[1.04;1.10].

As platelet (PLT) count increases, the ROD decreases, irrespectively of age, sex of the patient and of other important ROD factors in this analysis. ROD was more than two and seven times higher, compared to the reference category in patients with the PLT count at the TOD of (50–100] G/L (HR[95%CI] = 2.66[1.11;6.35]) and [0–50] G/L (HR[95%CI] = 8.44[2.50;28.44]), respectively.

The ROD increases by more than 9% with an increase in the LUC of 0.1 G/L units, HR[95%CI] = 1.09[1.01, 1.16] and more than eightfold with a decrease in mean corpuscular volume (MCV) by 1.0 fmmol, HR[95%CI] = 0.12[0.02;0.82]. However, these two findings are not independent of other risk factors studied.

**Fig. 2:**
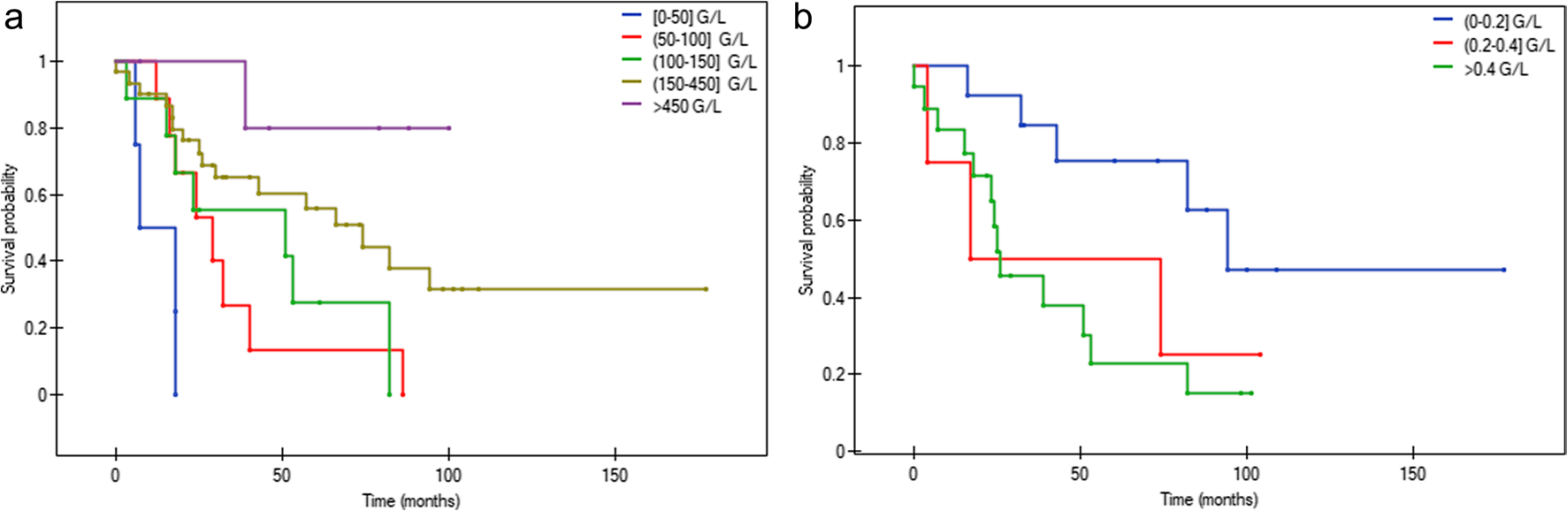
Kaplan–Meier analysis of the OS of PMF patients stratified according to: **a**) PLT count in the PB (*p* = 0.0002) **b**) LUC count in the PB (*p* = 0.0483). Abbreviations: OS – overall survival, PLT – platelets, PB – peripheral blood, LUC – large unstained cells

Kaplan–Meier analysis of the OS of the PMF patients stratified according to PLT and LUC count in the blood is presented in Fig. 2.

The median survival time was 0.5, 2.4, 4.3 and 5.7 years in patients with PLT count of [0–50], (50–100], (100–150] and (150–450] G/L at the TOD, and 7.8, 2.2 and 1.4 years in patients with the LUC count of [0.0–0.2], (0.2–0.4] and > 0.4 G/L at the TOD, respectively. Patients progressing during the follow-up to the AP or BP had lower PLT count and higher LUC count at the TOD than patients with B0 or B1 (Table 6).

**Table 6:** The relation between absolute PLT and LUC count in the PB at the TOD and disease progression to more advanced phases during the follow-up of the studied patients

Disease phase ^a^	PLT count (G/L), median, range	LUC count (G/L), median, range
BP	116 [70–185]	0.71 [0.48–0.89]
AP	129 [109–287]	1.48 [1.13–1.63]
B1	290 [137–409]	0.45 [0.12–2.23]
B0	252 [7–1258]	0.34 [0.04–1.34]

*Abbreviations*: *PLT* platelets, *LUC* large unstained cells, *AP* accelerated phase, *BP* blastic phase

^a^ The highest blast percentage in the peripheral blood/bone marrow (PB/BM) noticed during the disease outcome was used for the analysis: B0 [0–5%), B1 [5–10%), AP [10–19%] and BP ≥ 20%

### Factors influencing the PMF outcome by the ROC analysis

The analysis of the factors influencing the PMF outcome by the ROC analysis including age, *JAK2*V617F VAF, white blood cells, neutrophils, lymphocytes, monocytes, eosinophils, basophils, LUC, red blood cells count, hemoglobin concentration (Hb), hematocrit value (Hct), mean corpuscular volume, MCH, red cell distribution width (RDW), PLT, and mean platelet volume (MPV) at the TOD revealed that only age ≥ 54 years (AUC[95%CI] = 0.69[0.57;0.81], *p* = 0.0031) and the PLT count ≤ 185 G/L (AUC[95%CI] = 0.81[0.69;0.92], *p* = 0.0001) could serve as predictors of preterm death in patients with PMF (Table S5). However, it is worth noting that p-values obtained in the case of LUC (AUC[95%CI] = 0.67[0.48;0.86], *p* = 0.0891), Hb (AUC[95%CI] = 0.64[0.47;0.81], *p* = 0.0727), Hct (AUC[95%CI] = 0.65[0.48;0.81], *p* = 0.0653), RDW (AUC[95%CI] = 0.64[0.49;0.79], *p* = 0.0752, were close to statistical significance.

### Analysis of the impact of the mutational landscape at the TOD on the OS in PMF patients

**Fig. 3:**
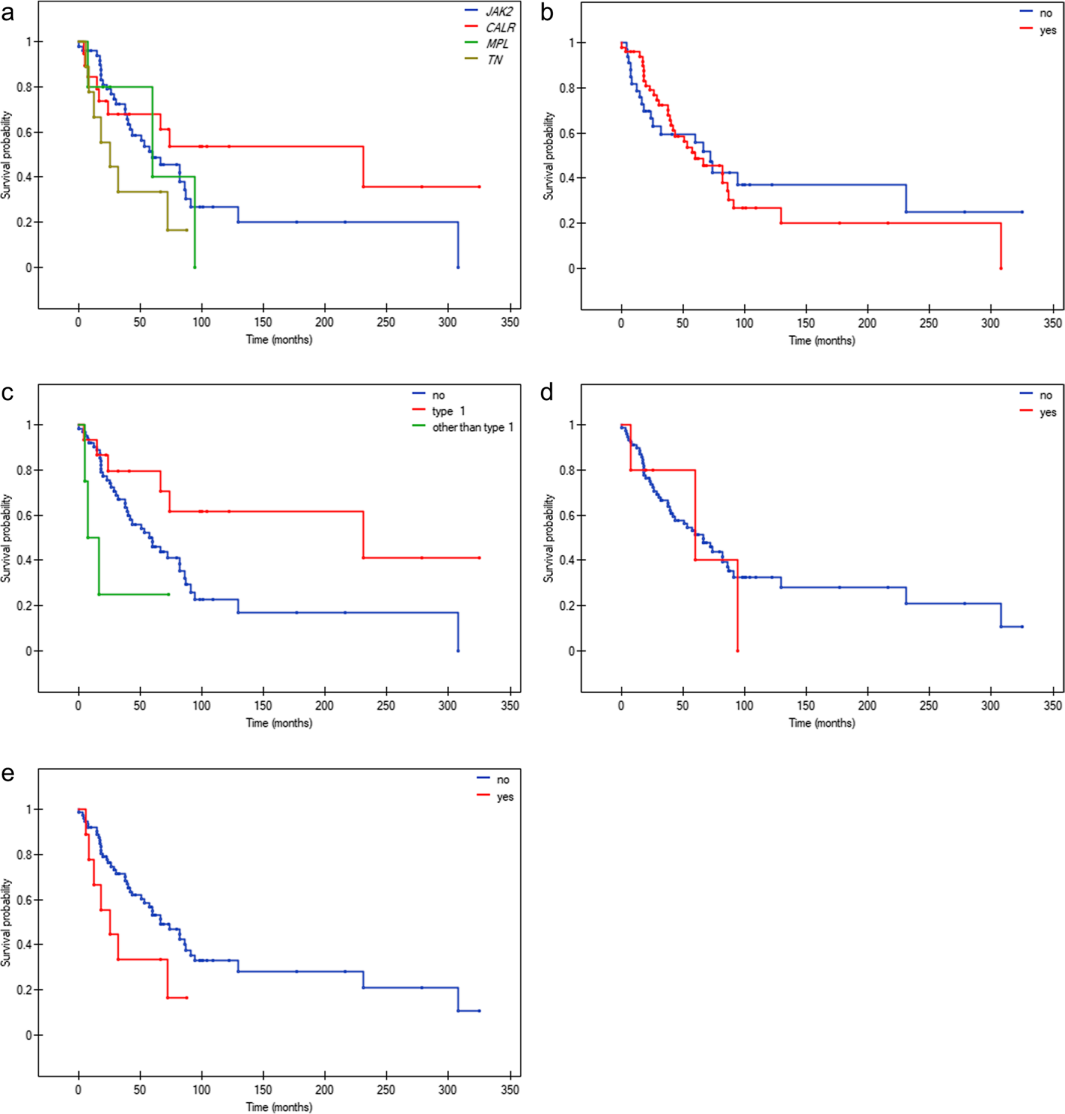
Kaplan–Meier analysis of the OS of PMF patients stratified according to: **a**) driver mutation type **b**) *JAK2*V617F mutation (*p* = 0.7388, ≤ 40^th^ month: *p* = 0.0915, yes/no: HR[95%CI] = 0.56[0.26;1.23] > 40^th^ month: *p* = 0.1487, yes/no: HR[95%CI] = 1.97[0.82;4.75]) **c**) *CALR* mutation type positivity (*p* = 0.0155, type 1/no: HR[95%CI] = 0.41[0.22;0.78], other than type 1/no: HR[95%CI] = 2.48[0.36;17.02], other than type 1/type 1 HR[95%CI] = 6.03[0.84;43.50]) **d**) *MPL* mutation presence (p = 0.7307) **e**) TN status (*p* = 0.0772)

**Fig. 4:**
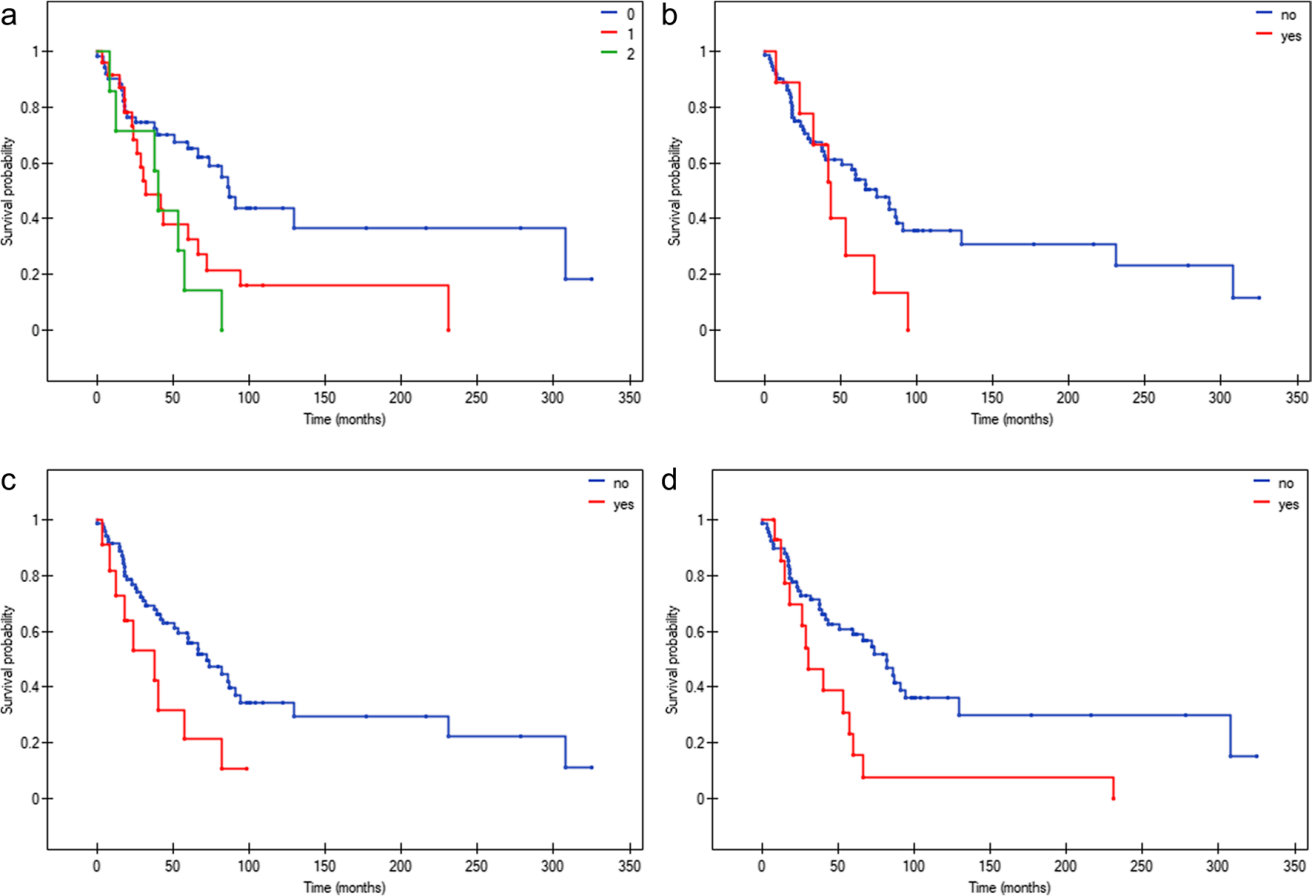
Kaplan–Meier analysis of the OS of PMF patients stratified according to their HMR variant type. Due to a small number of patients in the case, data concerning *U2AF1* S34, *IDH2*, *IDH1* are not shown. **a**) number of HMR variants detected (*p* = 0.0044, 1/0: HR[95%CI] = 2.12[1.10;4.09], 2/0: HR[95%CI] = 3.04[0.97;9.51]) **b**) *SRSF2* variant presence (*p* = 0.1123, ≤ 40^th^ month: *p* = 0.3599, yes/no: HR[95%CI] = 0.59[0.22;1.58] > 40^th^ month: *p* = 0.0001, yes/no: HR[95%CI] = 5.57[0.83;37.13]) **c**) *U2AF1*Q157 variant presence (*p* = 0.0305, HR[95%CI] = 2.15[0.82;5.61]) **d**) *ASXL1* variant presence (*p* = 0.0049, HR[95%CI] = 2.37[1.04;5.42])

Kaplan–Meier curves showing the OS of PMF patients according to individual categorical data are presented in Figs. 3 and 4. Among the driver mutations status, only *CALR* type 1 positively influenced the OS of PMF patients (Fig. 3c). The *JAK2*V617F, *MPL* and *CALR* gene mutations other than type 1 had no impact on the OS of the studied PMF patients***.*** However, the OS was negatively affected by the presence and number of coexisting HMR variants (Fig. 4a). Among separately analyzed HMR variants, the presence of *U2AF1*Q157 and *ASXL1* variants resulted in shorter OS (Fig. 4c, d). Interestingly, among 15 *CALR* type 1 positive patients, 4 out of 4 with a coexisting HMR variant and 2 out of 11 without a coexisting HMR variant died, respectively (Fig. 1 and Table S2*p* = 0.0110).

For variables that did not meet the proportional hazards assumption, the analysis was repeated in subgroups defined by the first quartile of survival time, i.e. 40 months – around which the curves intersected (≤ 40 months and > 40 months) – this relates to *JAK2*V617F and *SRSF2* status. Until the 40^th^ month of follow-up there was no difference in the OS depending on the *SRSF2* status. After the 40^th^ month of follow-up, the presence of the *SRSF2* mutation is associated with an unfavorable outcome (*p* = 0.0001) (Fig. 4b). Similarly, the impact of the *JAK2*V617F mutation changes after the 40^th^ month of follow-up and is associated with worse OS, however, it is not statistically significant (Fig. 3b).

### The analysis of the risk of unfavorable PMF outcome

The distribution of the different risk groups classified according to the IPSS and MIPSS70 scales is presented in Supplementary Fig. 1. The analysis showed similar frequency of patients at the high risk of unfavorable disease outcome at TOD (28 vs 31% according IPSS and MIPSS70 scales, respectively). However, contrary to the IPSS assessment, none of the patients studied was stratified as low risk when the MIPSS70 prognostic scale was used.

## Discussion

*JAK2*V617F expression in MPN Ph- patients is associated with strong hypermutable state and genomic instability of hematopoietic stem cells. Recent data indicate that in most of the MPN Ph- patients non-driver mutations are present already at the TOD. Their presence is now considered an unfavorable risk factor for the prognosis (Lundberg et al. 2014; Bartels et al. 2021a). Consistently with other reports, we confirmed that the presence of HMR variants was associated with significantly reduced OS (Guglielmelli et al. 2014; Lundberg et al. 2014; Patel et al. 2015). It was especially evident in the case of HMR co-existence in individual patients (5-year survival: 64%, 28%, 14% in the case of 0, 1, 2 HMR variants detected, respectively) and increased risk of progression of PMF to the AP and BP about 7 times.

According to GIPPS, which was reported to outperform the clinical based scoring system (Kuykendall et al. 2019), *CALR* type 1/type 1-like mutation are considered “good” risk factors for the OS, whereas *ASXL1*, *SRSF2* and *U2AF1*Q157 are unfavorable ones. These observations were also confirmed in our study. Moreover, the *ASXL1* and/or *U2AF1* mutations positivity at the TOD was associated with higher risk of progression to more advanced PMF phases. This is in line with the results of a meta-analysis covering 1393 PMF patients carrying the *ASXL1* mutation recently published by Wang et al., confirming adverse prognostic impact of *ASXL1* on the OS (Wang et al. 2021). On the contrary, in a study by Bartels et al., *ASXL1* mutation positivity was not associated with later PMF progression to the BP. However, the limitation of the above-mentioned study is the inclusion of other categories of MPN Ph- patients (PV, MPN unclassified) into the analysis (Bartels et al. 2021b). Other studies of PMF patients showed the negative impact of the *SRSF2* mutation presence on the risk of leukemic transformation and OS (Vannucchi et al. 2013; Vallapureddy et al. 2019; Bartels et al. 2021a). Our results did not confirm such association in terms of the blast phase transformation risk. However, we found a negative impact of the *SRSF2* mutation positivity on the OS after the 40^th^ month after diagnosis. The interpretation of such a phenomenon is difficult and requires further studies.

The analysis of the CBC results at the TOD showed an unexpected, hitherto undescribed, association between the LUC count in the blood and the prognosis in the studied patients with PMF. LUC are reported as a part of differential count in hemogram results and are not classified in the subgroups of leukocytes, like neutrophils, monocytes, eosinophils, lymphocytes, and basophils. LUC reflect the peroxidase-negative cells population in the peripheral blood and refer to large lymphocytes, virocytes, plasma cells, hairy cells, peroxidase-negative blast cells and hematopoietic progenitor cells (Thirup 1999). LUC count was found to be a useful diagnostic parameter in the case of clinical suspicion of acute leukemias (Rabizadeh et al. 2015), HIV infection (Vanker and Ipp 2014) and *Aspergillosis* (Cakir et al. 2018), in predicting hematological response to recombinant human granulocyte colony-stimulating factor (rHu-G-CSF) (Bononi et al. 2009) or successful collection of stem cells in case of progenitor cells mobilization to peripheral blood before autologous stem cell transplantation (Merter et al. 2022). In patients treated with chemotherapy, the absolute number of LUC was positively correlated with the absolute number of blasts and CD34 + cells in the prenadir and postnadir phases (Bononi et al. 2001). Unfortunately, there is no data concerning LUC count in the PB in patients with MPN Ph-. In our study, LUC count at the TOD was negatively correlated with the ROD and OS. Although LUC refer to different types of cells, it was recently documented that 1) the absolute LUC count is positively correlated with peripheral CD34 + cells content (Merter et al. 2022), 2) LUC are very likely to represent malignant PMF-initiating cells (Saito et al. 2022). LUC count appears to be equivalent to the content of abnormal cells in the PB; perhaps an analysis of this parameter could be a screening method, instead of time-consuming manual blood smear assessment or expensive flow cytometry. It should be noted, however, that flagging for blasts and immature granulocytes showed moderate sensitivity and specificity, depending on the type of the apparatus used (Meintker et al. 2013).

The PB and BM blast cells content at the TOD is an important factor influencing prognosis in patients with PMF. Masarova et al. demonstrate that PB blast percentages offer an additional prognostic value in patients who have < 5% blasts in the BM. They also suggest that both PB and BM blasts ≥ 5% might be considered similar to unfavorable karyotypes or HMR mutations (Masarova et al. 2020). According to Huang et al., peripheral blood blast percentage ≥ 3% at the TOD is a strong and independent predictor of leukemic transformation risk (Huang et al. 2008).

Thrombocytopenia (PLT < 100 G/L) is a negative prognostic marker according to DIPSS-Plus (Gangat et al. 2011). Importantly, our results show that a cohort with severe thrombocytopenia (PLT < 50G/L) at the TOD has even worse outcome than a cohort with moderate one (PLT 50–100 G/L), both regarding the disease phase and OS. These observations are directly in line with previous findings by Hernandez et al. and Masarova et al. (Masarova et al. 2018; Hernández-Boluda et al. 2018). In our opinion, it may be more appropriate to create a separate, higher score category in the prognostic scales to better define the risk of unfavorable PMF outcome in patients with severe thrombocytopenia. The proposal of such a strategy is based on our preliminary results confirming over three times higher risk of death and shorter median survival time (7 vs. 29 months) in PMF patients with the platelet count < 50 G/L at the TOD, in comparison to individuals with the PLT count between 50–100 G/L.

## Conclusion

Our analysis led to the following conclusions: in PMF patients at the TOD 1) the presence of HMR variants, especially combined, is associated with an increased risk of progression to the AP and BP, and shorter OS, 2) severe thrombocytopenia confers worse prognosis than the moderate one, 3) LUC count is closely related with the disease phase, and associated with the ROD and OS. In our opinion, the incorporation of the LUC count at the TOD in the risk assessment algorithms seems to be reasonable and useful from the clinical point of view. Complex, laboratory and genetic evaluation may help in early identification of PMF patients with worse prognosis due to high risk of disease progression to more advanced phases.

## Limitations of the study

This study has several limitations. Due to different types of the apparatus used in the centers participating in the study, a detailed CBC analysis in terms of LUC was limited to 35 patients only (Table S6). Moreover, the molecular PMF patients assessment was performed for HMR variants presence only and with the help of standard molecular techniques. Despite these limitations, we hope that the results presented by us will initiate further studies on clinical and prognostic significance of HMR variants and low platelet count in the PB at the TOD in PMF patients. We realize that prognostic significance of LUC count in the PB at the TOD in PMF patients should be confirmed in subsequent study in larger group of patients and CBC analyzers reporting LUC as a separate population in differential.

## Supplementary Information

Below is the link to the electronic supplementary material.

Supplementary file1 (DOCX 36 KB)

Supplementary file2 (XLSX 56 KB)

## Data Availability

The datasets generated and analysed during the current study are available from the corresponding author on request.
