# Supplementary material for: High molecular risk variants, severe thrombocytopenia and large unstained cells count affect the outcome in primary myelofibrosis
Source: J Appl Genet. 2023 Jul 29;64(3):479–91. doi: 10.1007/s13353-023-00771-x (PMC10457229; doi:10.1007/s13353-023-00771-x)
Supplement: Supplementary file 1 — Supplementary file1 (DOCX 36 KB) [file 13353_2023_771_MOESM1_ESM.docx]

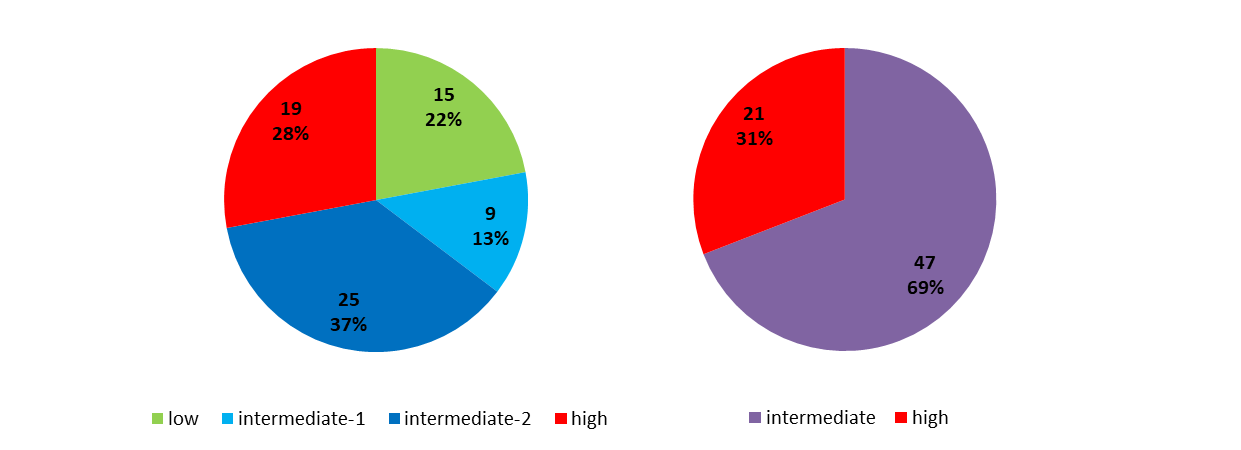
Supplementary Figure 1. The distribution of the different prognostic risk groups according to IPSS (A) and MIPSS70 (B) scales in analyzed patients with primary myelofibrosis*

A) B)

*the number of the evaluated cases is limited to 68 patients, because 14 out of 82 patients were older than 70 years. Due to the incomplete karyotype data, the other molecular scales (ie. MIPSS70+, MIPSS70+ v2.0 and GIPPS) could not have been applied.
